# Investigation of Formulations on Pyrene-Based Anodized-Aluminum Pressure-Sensitive Paints for Supersonic Phenomena

**DOI:** 10.3390/s22124430

**Published:** 2022-06-11

**Authors:** Kazuma Yomo, Tsubasa Ikami, Koji Fujita, Hiroki Nagai

**Affiliations:** 1 Institute of Fluid Science, Tohoku University, Sendai 980-8577, Japan; yomo.kazuma.p7@dc.tohoku.ac.jp (K.Y.); fujita.koji@tohoku.ac.jp (K.F.); nagai.hiroki@tohoku.ac.jp (H.N.); 2 Department of Aerospace Engineering, Tohoku University, Sendai 980-8577, Japan

**Keywords:** pressure-sensitive paint (PSP), pressure sensitivity, temperature dependency, luminescent intensity, time response

## Abstract

Pressure-sensitive paint (PSP) is an optical sensor that can measure global pressure distribution by using the oxygen quenching of dye molecules. In particular, anodized aluminum pressure-sensitive paint (AA-PSP) exhibits a fast time response. AA-PSP has been used in unsteady measurements at supersonic and transonic speeds, such as on the surface of a transonic free-flying sphere or the wall of a shock tube when the shock wave passes. To capture such ultrafast phenomena, the frame rate of the camera must be sufficiently fast, and the exposure time must be sufficiently short. Therefore, it is desirable that the AA-PSP exhibits bright luminescence, high-pressure sensitivity, and fast response time. This study focused on pyrene-based AA-PSPs and investigated their characteristics, such as luminescence intensity and pressure sensitivity, at different anodization times, dipping solvents, and dipping concentrations. Furthermore, a time-response test using a shock tube was conducted on the brightest AA-PSP. Consequently, the time for a 90% rise in pressure was 2.2 μs.

## 1. Introduction

Pressure is one of the most important physical characteristics in fluid dynamics. In particular, accurate measurements of surface pressure distributions on automobiles, high-speed rails, and aircraft help us to understand not only the aerodynamic forces acting on them, but also their stabilities and other phenomena. Pressure-sensitive paint (PSP) has attracted attention as a tool for the optical measurement of global pressure distribution using the oxygen quenching of dye molecules [1]. Non-contact measurement is one of the advantages of PSP. Probes, such as pressure transducers, must be attached to the test model directly, which can complicate the measurement system and interfere with the flow field. However, PSP only needs to coat the model surface. In addition, the pressure distribution can be measured optically by capturing the luminescence with CMOS or CCD cameras, owing to the fluorescence and phosphorescence of dye molecules in PSP. Therefore, PSP can easily be applied to models with complex shapes [1].

A PSP with a fast time response is required to capture pressure fluctuations under supersonic and transonic conditions—such as shock wave phenomena—without time delay [2,3,4]. The oxygen permeability of the model surface and the luminescence lifetime of the dye molecules are the two parameters related to the time response of the PSP. The better the oxygen permeability of the model surface, the faster the response time because PSP uses oxygen quenching. Good oxygen permeability is also useful in cryogenic wind tunnel tests where the oxygen partial pressure in the test gas is low [5,6,7,8]. The luminescence lifetime is the duration of the luminescence caused by the transition of the dye molecules from the excited state to the ground state. The shorter luminescence lifetime suggests that more accurate pressure fluctuations can be captured without a time delay.

Some studies have developed sprayable fast-PSPs, mixing small particles with a polymer to form the porous binder [9,10,11,12,13,14]. However, Anodized Aluminum-PSPs (AA-PSP) have the potential to achieve a faster time response because of the high oxygen permeability of the porous surface [15,16,17,18,19]. AA-PSP is fabricated by adsorbing a dye molecule onto an anodized aluminum model using the dipping method, AA-PSP exhibits good oxygen permeability, owing to the porous surface of AA-PSP and allows for fast time response. AA-PSPs have been employed for the pressure measurements in the transonic and supersonic flows [20,21,22,23,24,25]. Hangai et al. investigated the relationship between the type of electrolyte for anodization and the pore size of the model [26]. The results showed that relatively large pores approximately 100 nm in diameter were formed when phosphoric acid was used, and the response time was microsecond-order. Furthermore, Numata et al. investigated the relationship between the electrolyte temperature and the pore size when phosphoric acid was used as the electrolyte for anodization [18]. The largest pore size was observed when the electrolyte temperature was 30 °C. Moreover, the time for a 90% rise in pressure of their AA-PSP is 0.81 μs, the fastest time response to date. They have also succeeded in capturing shock waves passing around a cylinder using the AA-PSP.

The dye molecules used in AA-PSP can be roughly divided into ruthenium-complexes [27,28,29,30], porphyrins [19,31], and pyrene [18,26,30]. A previous study reported that the luminescence lifetimes of the luminophores used in AA-PSP in the air were about 10^2^ ns for ruthenium complexes, 1–10^2^ ns for porphyrin compounds, and 10 ns for pyrene compounds [19]. Therefore, pyrene is suitable for ultrafast measurements. Hangai et al. and Numata et al. have also used pyrene [18,26].

It is necessary to shorten the exposure time of the high-speed camera to capture instantaneous phenomena using AA-PSP. However, sufficient luminescence cannot be measured in this case, and pressure-distribution images have a low signal-to-noise ratio (SNR). The use of image intensifiers is one of the typical approaches to amplifying the luminescence [18]. Also, recent research reported that the luminescence intensity could be enhanced by suppressing the thermal quenching [32]. However, the basic and direct solution is to develop the AA-PSP with a high luminescence intensity and pressure sensitivity by optimizing the formulation.

The objective is to develop an AA-PSP with high luminescence intensity and high-pressure sensitivity, as well as a fast response time. This study focused on pyrene sulfonic acid (PSA). PSA is often used as a dye molecule of the fast response PSP [2,31,33,34]. One of the benefits of using PSA is the short lifetime because the response time is limited by the lifetime. We investigated the optimal anodization time, type of dipping solvent, and dipping concentration. Moreover, the time-series images of the shock wave passing in front of the optimized AA-PSP flat plate were captured using a high-speed camera. The time response of the optimized AA-PSP was evaluated using the pressure distribution images in this study.

## 2. Principle of AA-PSP

**Figure 1:**
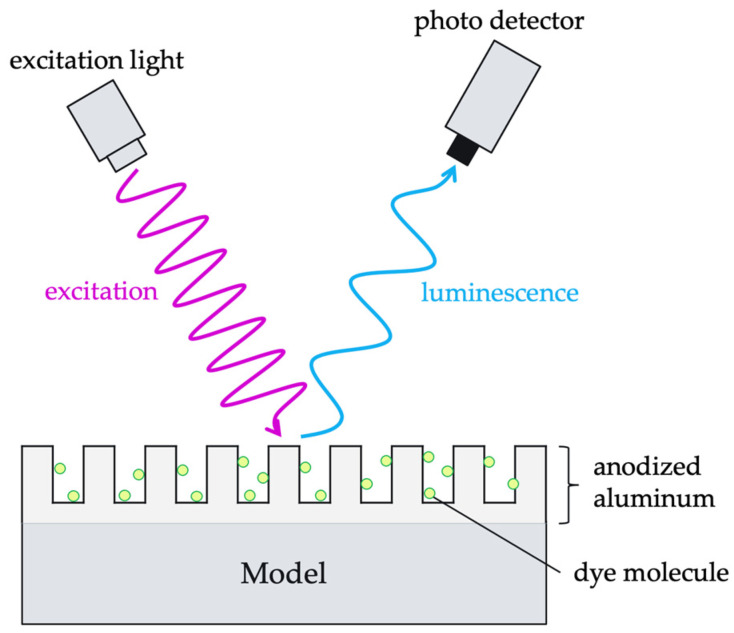
The principle and constitution of the AA-PSP measurement.

Figure 1 shows a schematic representation of the AA-PSP measurements. AA-PSP consists of dye molecules that act as pressure sensors and anodized aluminum that acts as a porous binder to adsorb the dye molecules [5,15]. When dye molecules on the AA-PSP surface are irradiated with excitation light of a specific wavelength, they acquire energy for the transition from the ground state to the excited state. The excited dye molecules emit energy in the form of luminescence and return to the ground state. However, their energy can be used to excite oxygen molecules present in the atmosphere. Therefore, higher oxygen concentration reduces the luminescence intensity. This phenomenon is known as oxygen quenching [1]. The luminescence intensity was lower at higher pressures and higher at lower pressures because the oxygen ratio was constant in the air. Therefore, the pressure distribution on the model surface can be measured by capturing the luminescence with a photodetector such as a camera.

The Stern–Volmer equation describes the relationship between the luminescence intensity and pressure in Equation (1). The graph corresponding to this equation is called the Stern–Volmer plot and is often used as a calibration curve [1].

(1) $$\frac{I_{\mathrm{ref}}}{I}=A\left(T\right)+B\left(T\right)\frac{P}{P_{\mathrm{ref}}}$$

where *I* is the luminescence intensity, *T* is the temperature, *A* and *B* are temperature-dependent coefficients, *P* is the pressure, and the subscript ref represents the reference state. In practice, the polynomial in Equation (2) is often used instead of Equation (1).

(2) $$\frac{I_{\mathrm{ref}}}{I}=\overset{N}{\underset{k=0}{\sum }}C_{k}\left(T\right){\left(\frac{P}{P_{\mathrm{ref}}}\right)}^{k}\;$$

Here, *k* is the degree of the polynomial, and *C_k_* (*k =* 0, 1, …, *N*) is the polynomial coefficients. *C_k_* is obtained by a calibration test.

## 3. Preparation of Test Piece

### 3.1. Formulations of PSP

In this study, we investigated the characteristics of PSA-based AA-PSP with different anodization times, dipping solvent types, and dipping concentrations. Phosphoric acid was used as the electrolyte for anodization. The dipping concentration *C* is defined in Equation (3).

(3) $$\;C\left(\mathrm{mM}\right):\frac{\mathrm{amount}\;\mathrm{of}\;\mathrm{dye}\;\left(\mathrm{mmol}\right)}{\mathrm{volume}\;\mathrm{of}\;\mathrm{solvent}\;\left(L\right)}\;$$

Table 1 summarizes the formulations used in this study. Three dipping solvents were compared: ethanol, dichloromethane, and their mixture in a 1:9 volume ratio. It should be noted that PSA was not completely dissolved in dichloromethane. On the other hand, PSA was completely dissolved in the mixture of ethanol and dichloromethane because PSA is well soluble in ethanol. In this comparison, the anodization times and dipping concentrations were fixed at 60 min and 0.1 mM, respectively. Furthermore, the anodization times and dipping concentrations of the mixed solvents were investigated.

### 3.2. Anodization

The test pieces were flat plates of aluminum alloy A5052 with a thickness of 1 mm. The effective area of each test piece was 40 mm × 40 mm, and unnecessary areas were masked with a Kapton tape.

First, the test pieces were pretreated by dipping them in NaOH solutions (0.3 wt%). This pretreatment removed unnecessary oil and other contaminants from the surface of the test pieces. They were then rinsed with distilled water, blown off with an air gun, and dried in a desiccator for approximately one day.

Next, the test pieces were anodized in a phosphoric acid solution (0.3 mol/L). The electrolyte temperature was fixed at 30 °C, and the current density was 12.5 mA/cm^2^. It should be noted that the current density was increased linearly to the target value over 5 min to avoid a sudden increase in the current density, which may cause the surface to burn. Then, they were rinsed with distilled water, and the excess water was blown off with an air gun.

After sufficient drying, the test pieces were dipped in phosphoric acid solutions (0.3 mol/L) at 30 °C for 10 min as a post-treatment, which removed the excess precipitates. Subsequently, the test pieces were rinsed with distilled water, the excess water was blown off with an air gun, and dried in a desiccator for approximately one day. The anodization conditions used are listed in Table 2.

### 3.3. Dipping

The dye molecules were adsorbed using the dipping method. First, the solvent and PSA were magnetically stirred in a beaker. In the case of mixed solvents, the solvents were mixed first, and then PSA was dissolved. After sufficient dissolution, the specimens were dipped into the solution that was controlled at 20 °C. The dipping time was set to 1 h. Subsequently, excess solution was blown off using an air gun and dried in a desiccator.

## 4. Experimental and Analytical Methods

### 4.1. Calibration System

**Figure 2:**
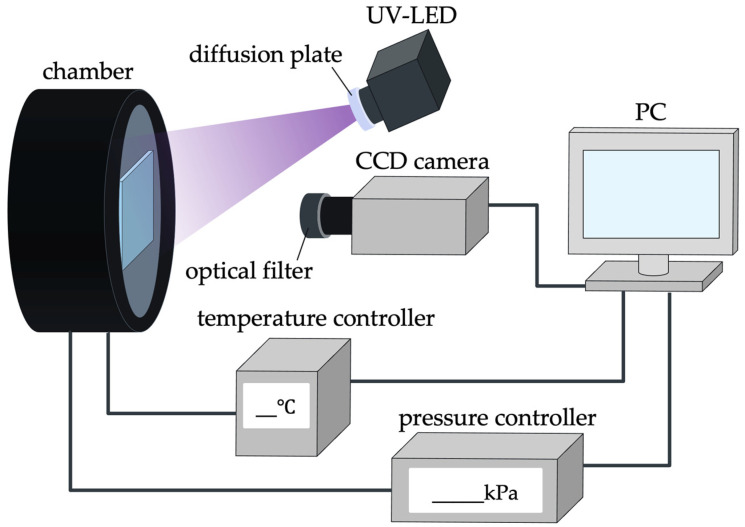
A schematic of the calibration system for PSP.

Figure 2 shows a schematic of the PSP calibration system used in this experiment. Details of the calibration system can be found in [35]. The pressure in the chamber was controlled by using a pressure controller (PACE6000; Baker Hughes, Houston, TX, USA). The PSP test piece was placed on a Peltier unit (PU-50WS, Takagi MFG., Hitachinaka, Japan) in the chamber. The surface temperature of the test piece was measured using a resistance temperature detector (NFR-CF2-0305-20-100S-1-1000TF(PTFE13)-A-3-M4Y, Netsushin, Miyoshi, Japan) and by controlling the Peltier unit. The optical window of the chamber is composed of quartz glass.

A UV-LED (SOLIS-365C, THORLABS, Newton, NJ, USA) was used as the excitation light source for PSP. The central wavelength of this excitation light was 365 nm, and the bandwidth was 10 nm. A diffusion plate was attached to the light source. The distance between the light source and the PSP test piece was approximately 300 mm. A 16-bit CCD camera (C4742-98 KAG2, Hamamatsu Photonics, Hamamatsu, Japan) was used to detect the PSP luminescence. A lens (Nikkor 50 mm f/1.4, Nikon, Tokyo, Japan) was attached to this camera, and an optical short-pass filter (SV0550 long-wavelength cutoff filter VIS 550 nm 50 × 50, ASAHI SPECTRA) and optical long-pass filter (TS high-performance OD 4.0, long-pass filter 400 nm 50 mm, Edmund Optics, Barrington, NJ, USA) were installed in front of the lens.

### 4.2. Calibration Test

The calibration test was performed using the calibration system described in Section 4.1. Images of the AA-PSP surface at different pressures in the 10–100 kPa were acquired using a CCD camera. The temperature was maintained at 17 °C.

To create the Stern–Volmer plot, *I*_ref_/*I* for each pressure condition was calculated in 100 × 100 pixels on the test piece, which corresponds to 37 mm^2^, and then averaged. The reference pressure was set as 100 kPa. The standard deviation of the calculated area is shown as the error bar.

It is assumed that the photodegradation followed exponential attenuation to the number of irradiations of the excitation light, as shown in Equation (4).

(4) $$\;I\left(t\right){=I}_{0}\exp(-\lambda t)$$

Here, *I*_0_ is luminescence intensity at *t* = 0, *λ* is the photodegradation rate, and *t* is the time or number of irradiations. The images were acquired under the same pressure and temperature conditions at the beginning and end of the calibration test. The photodegradation rate λ can be calculated by comparing the luminescence intensities of these two images. In this study, the luminescence intensity of each image was corrected based on its photodegradation rate.

In addition, the images were acquired under the same pressure and temperature conditions at the beginning and end, and the photodegradation rates between the two images were calculated. The luminescence intensity of each image was corrected based on its photodegradation rate.

The local pressure sensitivity *S_P_* was calculated by Equation (5).

(5) $$S_{P}\left(\%/\mathrm{kPa}\right)=-\left(\frac{dI\left(P\right)}{dP}\right)\frac{1}{I\left(P\right)}\times 100\;$$

The luminescence intensity was interpolated using a spline approximation, and the pressure sensitivity was calculated by differentiating the function.

### 4.3. Measurement of Luminescence Intensity

The luminescence intensities of the test pieces with different formulations were compared. The temperature was fixed at 17 °C, and the pressure was changed to the range of 10–100 kPa. The measurements were conducted within 72 h after the sample preparation. In addition, the samples were dried in a desiccator in a dark room. The camera settings and excitation light output were the same for all test pieces. However, if a pixel was saturated, the camera exposure time was changed and later corrected to match the other conditions. The luminescence intensity was averaged at 100 × 100 pixels for each test piece. The standard deviation of the averaged range is the error bar.

### 4.4. Shock Tube

**Figure 3:**
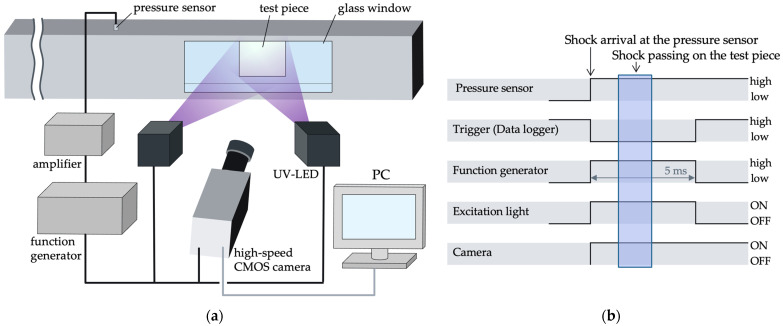
Pictures of the shock tube. (**a**) A schematic of components and their connections. (**b**) A timing chart.

Figure 3a shows the schematic of the shock tube used in this experiment. This shock tube is a diaphragm-type, and a Mylar film separates the high-pressure and low-pressure sections. The square cross-section shock tube had a glass window (BK7), and a test piece of 80 mm × 80 mm was attached to the opposite side of the glass window. Two UV-LEDs (SOLIS-365C, THORLABS, Newton, NJ, USA) were used as excitation lights. A high-speed video camera (Phantom v2011, Vision Research Inc., Wayne, NJ, USA) was used to capture the shock wave passing in front of the test piece. A lens (Nikkor 50 mm f/1.4, Nikon, Tokyo, Japan) was attached to the camera, and an optical short-pass filter (SV0550 long-wavelength cutoff filter VIS 550 nm 50 × 50, ASAHI SPECTRA, Tokyo, Japan) and optical long-pass filter (TS high-performance OD 4.0, long-pass filter 400 nm 50 mm, Edmund Optics, Barrington, NJ, USA) were installed in front of the lens.

A pressure transducer (603B1, KISTLER, Winterthur, Switzerland) was placed at 400 mm in front of the test piece to detect the passing shock wave as an electrical signal, and a trigger signal was input to the camera via a charge amplifier (Type5007, KISTLER, Winterthur, Switzerland) and multifunction generator (WF1974, NF Corporation, Yokohama, Japan). At the same time, the multifunction generator provides a 5 ms rise signal to the UV-LEDs, so that the UV-LEDs irradiate the test piece only when the shock wave passes in front of it. This process enabled the suppression of the photodegradation of the test piece. The timing chart is shown in Figure 3b.

### 4.5. Measurement of Time Response

The time-response test was performed using a shock tube, as shown in Figure 3. Test pieces with anodization times of 30, 60, and 90 min were tested to evaluate differences in the time response. The dipping concentration was fixed at 0.1 mM.

The low-pressure area where the test piece was attached during the test was decompressed to 30 kPa. A shock wave with a Mach number of 1.4 was passed through it. The 30 kPa pressure difference can be measured at the shock wave because the pressure ratio before and after the shock wave is approximately 2. Furthermore, the pressure difference was discontinuous. Therefore, the time response can be evaluated by measuring the rise time of the PSP to the shock wave passing through. 

**Figure 4:**
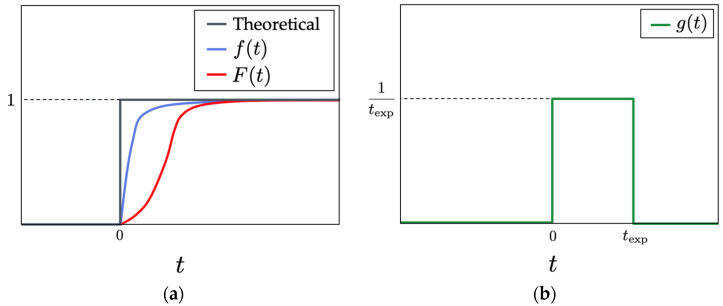
The pressure fluctuations during a shock wave, passing through. (**a**) Time response curves. (**b**) The function *g*(*t*) used for the convolution of *f*(*t*).

Figure 4a shows a schematic of the theoretical pressure rise of the shock wave, the pure response curve *f*(*t*) of the PSP, and the response curve *F*(*t*) of the PSP, including the effect of the camera exposure time. The pure response curve of PSP exhibits a delay in the discontinuous pressure change of the shock wave. The function is approximated by the first-order lag system in Equation (6):

(6) $$\;f\left(t\right)=1\;-\exp\left(-\frac{t}{\tau }\right)\;$$

In addition, the effect of exposure time *t*_exp_ appears when acquiring images. Therefore, it is necessary to consider the effect of the exposure time *t*_exp_ when fitting the curve to obtain the time constant *τ* in Equation (6) from the measured curve. Then, a convolution integral of the pure response curve *f*(*t*) is performed, as in Equation (7), using the square pulse function *g*(*t*) shown in Equation (8) and Figure 4b. Thus, the actual response curve *F*(*t*) was obtained.

(7) $$\;F\left(t\right)=\int_{0}^{t}f\left(t\;-\;u\right)g\left(u\right)du$$

(8) $$\;g\left(t\right)=\{\begin{array}{c} \frac{1}{t_{\exp}}\;\;\;\;\left(0\;\leq \;t\;\leq {\;t}_{\exp}\right)\\ \;\;\;\;\;\;0\;\;\;\;\;\;\left({t\;<\;0,\;t}_{\exp}\;<\;t\right) \end{array}\;$$

For practical use, the time constant *τ* was used as a fitting parameter, and the function *F*(*t*) was fitted for the experimental data by the least square method [18]. As a result, the most appropriate time constant *τ* was obtained. In this study, the acquired pressure distribution was converted into a time-series pressure history using the shock wave velocity. The shock wave velocity was measured by the pressure transducers. The initial time was defined as the time when the pressure began to increase in the pressure history.

The time constant *τ* in the first-order lag system in Equation (6) can be determined by fitting *F*(*t*) to the experimental curve. The 90% rise time in Equation (9) is often used to evaluate the time response of PSP.

(9) $$\tau_{90\%}=\tau \ln10$$

## 5. Results and Discussion

### 5.1. Pressure Sensitivity

In this section, we discuss the results of the calibration tests. The Stern–Volmer plots show the pressure on the horizontal axis and luminescence intensity ratio on the vertical axis, normalized by the luminescence intensity at a pressure of 100 kPa. The temperature was maintained at 17 °C. The pressure sensitivities were calculated using Equation (5).

#### 5.1.1. Different Dipping Solvents

**Figure 5:**
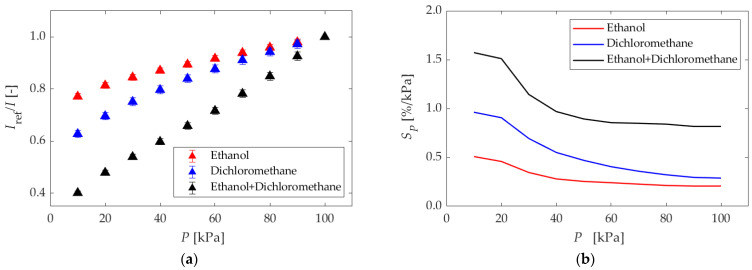
The results in different dipping solvents. (**a**) The Stern–Volmer plots. Each reference condition is *P* = 100 kPa. (**b**) The pressure sensitivities.

Figure 5 shows the results for different dipping solvents. Figure 5a shows the Stern–Volmer plot. The slope of the luminescence intensity ratio was larger for the mixed solvent than for a single solvent, ethanol or dichloromethane. Figure 5b shows the pressure sensitivities obtained using Equation (5). The mixed solvent exhibited the best pressure sensitivity at all pressures. Therefore, it can be concluded that the mixture of ethanol and dichloromethane is the optimal solvent in the present study. The pressure sensitivities decreased with increasing pressure for the dichloromethane solvent at all pressures. The pressure sensitivities also decreased with increasing pressure for the ethanol and mixed solvents in the range of 10–60 kPa. However, the pressure sensitivity was almost constant above 60 kPa for these solvents. 

#### 5.1.2. Different Anodization Time and Different Dipping Concentrations

**Figure 6:**
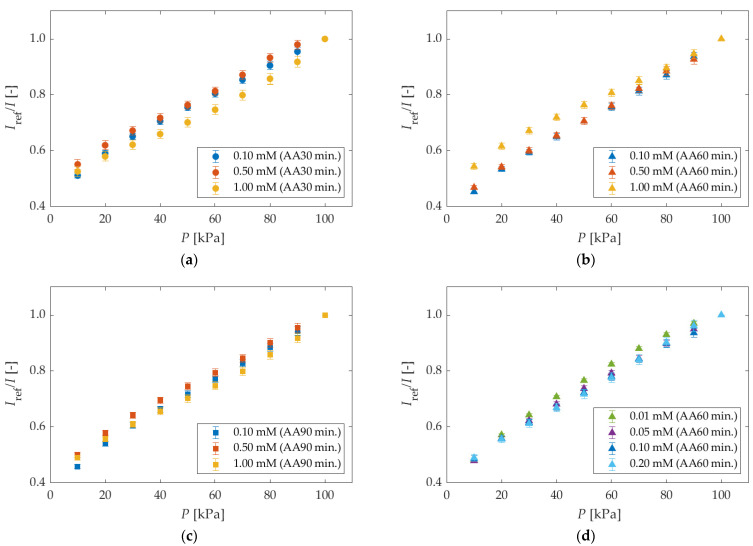
The Stern–Volmer plots in different anodization times and different dipping concentrations. Each reference condition is *P* = 100 kPa. AA means the anodization time in the legend. (**a**) anodization time of 30 min; (**b**) anodization time of 60 min. (more than 0.10 mM); (**c**) anodization time of 90 min; (**d**) anodization time of 60 min (less than 0.20 mM).

Figure 6 shows Stern–Volmer plots for different anodization times and different dipping concentrations. The trends for all Stern–Volmer plots are similar. However, slight nonlinearities appeared at the dipping concentration of 1.00 mM at all anodization times.

**Figure 7:**
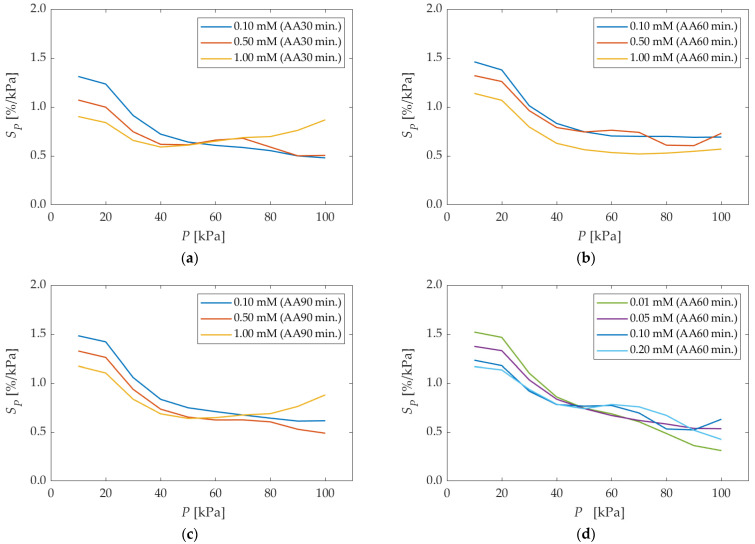
The pressure sensitivities in different anodization times and different dipping concentrations. AA means the anodization time in the legend. (**a**) anodization time of 30 min; (**b**) anodization time of 60 min (more than 0.10 mM); (**c**) anodization time of 90 min; (**d**) anodization time of 60 min (less than 0.20 mM).

Figure 7 shows the pressure sensitivities for different anodization times and dipping concentrations. The pressure sensitivities basically decreased with increasing pressure for all the results, and they were 0.5–1.5%/kPa for the most part. However, the curves at the dipping concentration of 1.00 mM took a minimum value of 40–70 kPa for all anodization times. This is a slightly different tendency compared to other dipping concentrations.

### 5.2. Luminescence Intensity

**Figure 8:**
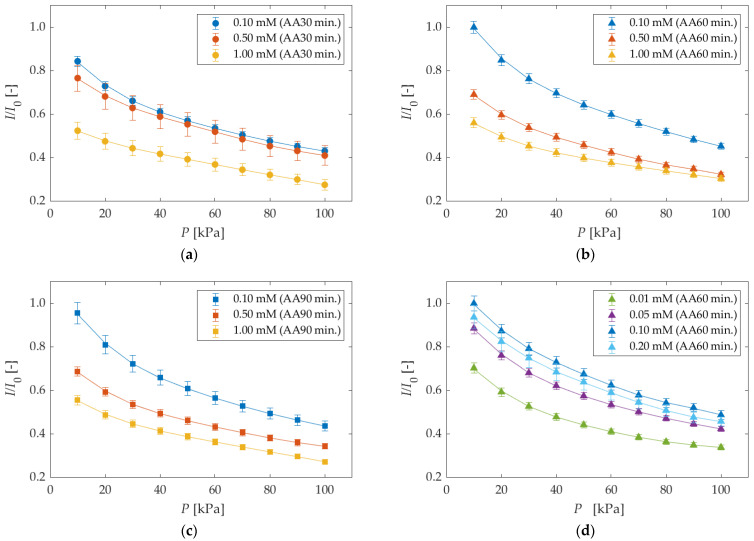
The luminescence intensities in different anodization times and different dipping concentrations. AA indicates anodization time in the legend. All luminescence intensities were normalized with the luminescence intensity at 100 kPa at 0.10 mM (AA60 min). The solid lines represent the spline approximation for each plot. (**a**) anodization time of 30 min; (**b**) anodization time of 60 min (more than 0.10 mM); (**c**) anodization time of 90 min; (**d**) anodization time of 60 min (less than 0.20 mM).

Figure 8 shows the luminescence intensities for different anodization times and dipping concentrations. All luminescence intensities are normalized with the luminescence intensity *I*_0_ at 0.10 mM (AA60 min). The luminescence intensities tended to decrease with increasing pressure in all results. The 0.10 mM (AA60 min) test piece showed the largest luminescence intensity. The solid line represents the spline approximation. In practice, the pressure sensitivities shown in Figure 7 were calculated using this approximation and Equation (5).

**Figure 9:**
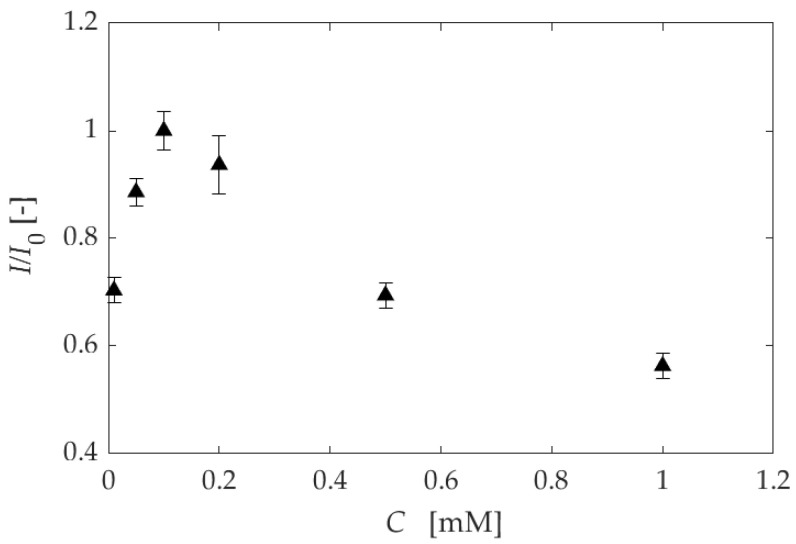
A comparison of the luminescence intensity against the dipping concentration at 10 kPa.

Figure 9 compares the luminescence intensity against the dipping concentration at 10 kPa. The intensity was normalized by the maximum value at 0.10 mM. The luminescence intensity increased with the concentration in the range of 0.01–0.10 mM. However, the luminescence intensity decreased in the range of 0.10–1.00 mM. The concentration quenching occurs in this dipping concentration range, and it can be concluded that 0.10 mM is the optimized dipping concentration in the range of the present study.

### 5.3. Time Response

**Figure 10:**
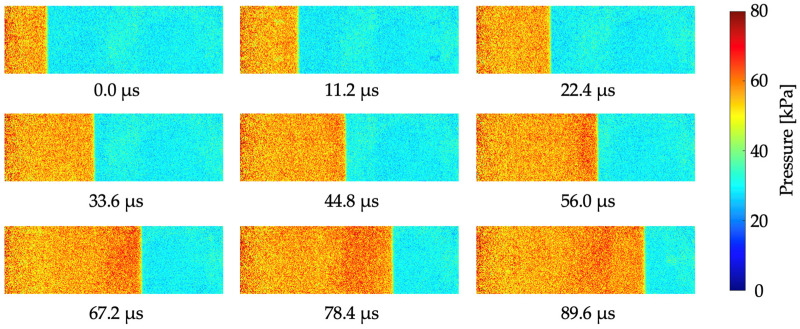
Time-series pressure distribution images of the 0.10 mM (AA60 min) test piece during the pass-through shock wave. The camera exposure time is 1.5 μs.

**Figure 11:**
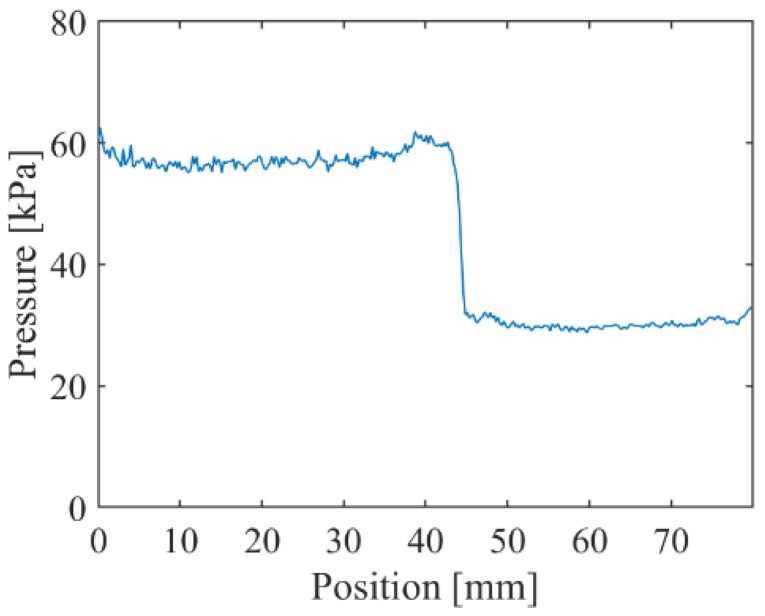
The pressure profile of the 0.10 mM (AA60 min) test piece at 56.0 μs.

Figure 10 shows the time series of the pressure distribution images as the shock wave passes in front of the 0.10 mM (AA60 min) test piece. For the calculation of the absolute pressure, the results of the calibration test in Figure 6 were used. The spatial resolution was 0.23 mm/pixel, and the camera exposure time was 1.5 μs. Figure 11 shows the pressure profile at 56.0 μs. The discontinuous pressure change in the shock wave was clearly observed in the pressure profile. The pressure for the high and low regions was about 60 kPa and 30 kPa, respectively. Therefore, the theoretical pressure change was accurately captured.

Table 3 summarizes the 90% rise time of pressure for each test piece. A spatial averaging was applied to the pressure distribution in the vertical direction in Figure 10. All results were calculated from fittings that considered the effect of camera exposure times. Therefore, the effects of exposure time were eliminated by Equation (7), and the calculated 90% rise time was shorter than the camera exposure time. The longer the anodization time, the slower the response time for all camera exposure times. The brightest test piece of 0.10 mM (AA60 min) showed an average response time of 2.2 μs. It should be noted that the faster response AA-PSP can be fabricated by reducing the anodization time. However, it may deteriorate the luminescence intensity, as shown in Figure 8.

## 6. Conclusions

This study investigated the pressure sensitivity, luminescence intensity, and time response of pyrene-based AA-PSP with different anodization times, dipping solvents, and dipping concentrations. The findings of this study are summarized as follows:

- Pressure sensitivity

The test pieces adsorbed PSA molecules with a mixed solvent of ethanol and dichloromethane showed higher pressure sensitivity than those with each solvent. The anodization time and dipping concentration of the mixed solvents were also investigated. The Stern–Volmer plots were linear in the range of 10–100 kPa. However, nonlinearity was observed at 1.00 mM dipping concentration. The pressure sensitivities basically decreased with increasing pressure for all cases.

- Luminescence intensity

The 0.10 mM (AA60 min) test piece showed the highest luminescence intensity. The luminescence intensities were considered to be small because the amount of PSA molecules was low when the dipping concentration was lower than 0.10 mM. On the other hand, the concentration quenching may occur above 0.10 mM. Therefore, the optimal dipping concentration is 0.10 mM in this study.

- Time-response test

The time for a 90% rise in pressure for the 0.10 mM (AA60 min) test piece with the highest luminescence intensity was measured to be 2.2 μs on average. The response time became slow as the anodization time increased. This study showed that a high-speed video camera could be used to visualize their time-series pressure distribution images. This result indicates the presented AA-PSP also had enough luminescence intensity for the microsecond-order camera exposure time.

## Figures and Tables

**Table 1:** Summary of formulations investigated in this study.

Anodization Time	Dipping Solvent	Dipping Concentration
30 min	Ethanol + Dichloromethane	0.10–1.00 mM ^1^
	Ethanol	0.10 mM
60 min	Dichloromethane	0.10 mM
	Ethanol + Dichloromethane	0.01–1.00 mM
90 min	Ethanol + Dichloromethane	0.10–1.0 mM

^1^ M: molar concentration (mol/L).

**Table 2:** A summary of the anodization process.

**Pretreatment**	Solution	NaOH aq (0.3 wt%)
	Dipping time	10 min
**Anodization**	Electrolyte	Phosphoric acid (0.3 mol/L)
	Current density	12.5 mA/cm^2^
	Temperature	30 °C
**Post-treatment**	Solution	Phosphoric acid (0.3 mol/L)
	Temperature	30 °C
	Dipping time	10 min

**Table 3:** The results of pressure rise time to reach 90%. The images were captured with the camera exposure times of 1.5 μs, 3.0 μs, 10.0 μs for each test piece.

	Test Piece 1	Test Piece 2	Test Piece 3
	AA30 min	AA60 min	AA90 min
*t*_exp_ = 1.5 μs	1.0 μs	2.5 μs	3.0 μs
*t*_exp_ = 3.0 μs	0.9 μs	2.8 μs	3.2 μs
*t*_exp_ = 10.0 μs	1.1 μs	1.4 μs	2.5 μs
Average $\;\tau_{90\%}$	1.0 μs	2.2 μs	2.9 μs

## Data Availability

Data sharing not applicable.
